# Distinguished Frontal White Matter Abnormalities Between Psychotic and Nonpsychotic Bipolar Disorders in a Pilot Study

**DOI:** 10.3390/brainsci15020108

**Published:** 2025-01-23

**Authors:** Takashi Shiroyama, Masayuki Maeda, Hisashi Tanii, Eishi Motomura, Motohiro Okada

**Affiliations:** 1 Department of Neuropsychiatry, Division of Neuroscience, Graduate School of Medicine, Mie University, 2-174 Edobashi, Tsu 514-8507, Mie, Japan; motomura@med.mie-u.ac.jp (E.M.); okadamot@med.mie-u.ac.jp (M.O.); 2 Department of Neuroradiology, Graduate School of Medicine, Mie University, 2-174 Edobashi, Tsu 514-8507, Mie, Japan; mmaeda@med.mie-u.ac.jp; 3 Center for Physical and Mental Health, Mie University, 1577 Kurimamachiya-cho, Tsu 514-8507, Mie, Japan; h-tanii@hac.mie-u.ac.jp; 4 Department of Health Promotion and Disease Prevention, Graduate School of Medicine, Mie University, 1577 Kurimamachiya-cho, Tsu 514-8507, Mie, Japan

**Keywords:** dysmyelination, oligodendrocyte, corpus callosum, corona radiata

## Abstract

Background/Objectives: Recent studies indicate extensive shared white matter (WM) abnormalities between bipolar disorder (BD) and schizophrenia (SZ). However, the heterogeneity of WM in BD in terms of the presence of psychosis remains a critical issue for exploring the boundaries between BD and SZ. Previous studies comparing WM microstructures in psychotic and nonpsychotic BDs (PBD and NPBD) have resulted in limited findings, probably due to subtle changes, emphasizing the need for further investigation. Methods: Diffusion tensor imaging measures were obtained from 8 individuals with PBD, 8 with NPBD, and 22 healthy controls (HC), matched for age, gender, handedness, and educational years. Group comparisons were conducted using tract-based spatial statistics (TBSS). The most significant voxels showing differences between PBD and HC in the TBSS analyses were defined as a TBSS-ROI and subsequently analyzed. Results: Increased radial diffusivity (RD) in PBD compared to NPBD (*p* < 0.006; *d* = 1.706) was observed in TBSS-ROI, distributed in the confined regions of some WM tracts, including the body of the corpus callosum (bCC), the left genu of the CC (gCC), and the anterior and superior corona radiata (ACR and SCR). Additionally, NPBD exhibited significant age-associated RD increases (R^2^ = 0.822, *p* < 0.001), whereas the greater RD observed in PBD compared to NPBD remained consistent across middle age. Conclusions: Preliminary findings from this small sample suggest severe frontal WM disconnection in the anterior interhemispheric communication, left fronto-limbic circuits, and cortico-striatal-thalamic loop in PBD compared to NPBD. While these results require replication and validation in larger and controlled samples, they provide insights into the pathophysiology of PBD, which is diagnostically located at the boundary between BD and SZ.

## 1. Introduction

BD is a severe psychiatric illness characterized by mood dysregulation, with recurrent depressive episodes and at least one manic or hypomanic episode, which is often associated with simultaneous psychotic symptoms [1]. Emotional domains including emotional intelligence, emotional face recognition, emotion-related decision-making, reward processing, and emotion regulation are affected in BD [2]. Cognitive deficits in BD are found even in euthymic state in verbal and nonverbal memory, attention, working memory, processing speed, and executive function [3,4]. Functional MRI (fMRI) studies [5,6,7,8,9,10] have elucidated that an altered functional network is associated with the impairment of emotional processes [5,7,9] and cognition [7], including social cognition [6,8], in BD. Alteration of the interhemispheric functional connectivity (FC) was reported in emotional processing related areas including sensorimotor area [11], the ventral prefrontal cortex (PFC), and insula cortex [12]. Abnormal activation pattern or FC in the fronto-limbic circuits [5,7,9], fronto-limbic-striatal network [13], thalamocortical network [14], and medial visual network [8] during emotion processing were also reported. In terms of cognition, altered activation in the dorsolateral PFC [15] during working memory tasks, the left superior and right inferior parietal lobule and left medial orbitofrontal cortex during cognitive tasks [7], and hyperactivation of orbitofrontal cortex during reward processing [5] were described. Concerning with social cognition, less activation in the posterior cingulate cortex during other- reflection tasks [6], deteriorated FC between ventromedial PFC and the amygdala in face recognition tasks [16], and less activation in the anterior cingulate to the theory of mind (ToM) tasks [17] were reported. Additionally, dysfunction of the default mode network (DMN), which is engaged in emotional processing and self-referential mental activity [18], has been elucidated in BD.

The DTI technique, based on the diffusion of water molecules in nerve fibers, was used to explore the alteration of WM microstructures, in which the values of the DTI measures, including FA, mean diffusivity (MD), axial diffusivity (AD), and RD, were evaluated. DTI measures are influenced by a variety of factors within WM tracts, such as the number of axons, axon density, axon diameter, and myelin thickness. FA is most commonly used as a marker of WM integrity, although it is a rather nonspecific marker of WM changes. Decreased FA is reported in a broad spectrum of diseases. MD provides bulk diffusivity, ignoring direction. Increased tissue water in edema increases MD, while increased MD is also reported to cause decreased integrity of WM tracts. While AD may be a more specific marker of axon damage, RD is affected by the degree of myelination [19,20].

While WM microstructures have been reported to be associated with cognition [4,15,21,22,23], emotional regulation [24,25], and functional networks [11,12,26,27], WM abnormalities have been established in the pathophysiology of BD [28,29,30,31,32,33]

Accumulated evidence indicates common, widespread WM abnormalities between BD and SZ [28,29,33,34,35,36,37,38,39,40,41]. However, although more than 50% of BD patients manifest psychotic symptoms in their lifetime [42], BD studies frequently include mixed subjects of PBD and NPBD [28,29,33,37,40]. While shared [34,38,39,43,44,45,46] and distinct [39,47] WM abnormalities between PBD and SZ have also been reported, the heterogeneity of WM microstructures of BD in terms of the presence of psychosis [33,46,48,49] is a critical issue for exploring the boundaries of BD and SZ, which has been a matter of debate since Kraepelin’s dichotomy [33,46,49,50,51,52].

According to previous studies [46,49,51,52], PBD is diagnostically located on the boundary between BD and SZ. It has been reported that PBD is distinguished from NPBD in neurocognition [53,54] and social cognition [55], in addition to emotional dysregulation [42,56]. Furthermore, functional [57,58] and structural [59,60] MRI and genetic studies [46,49] suggest a biological distinction between PBD and NPBD. As the neural basis for the above-mentioned differences, WM microstructural changes in PBD are, therefore, also assumed to be distinguished from NPBD.

However, a direct comparison of WM changes between PBD and NPBD has resulted in limited findings: decreased FA in the bCC [61], increased FA in the left uncinate fasciculus [62] in PBD, and no differences [29,40,63,64]. These limited results suggest that the differences in WM between PBD and NPBD are too subtle to be detected by previous methods.

Subtle dysmyelination causes cortical network dysfunction and catatonia-like symptoms in mice [65,66]. Subtle WM differences between PBD and NPBD can, therefore, be the key to elucidating the pathophysiology of simultaneous affective and psychotic symptoms in PBD [1,51]. We thus planned a pilot study based on the hypothesis that the most disrupted WM region in PBD compared to HC would exhibit more severe alterations than in NPBD, which has not been investigated in previous comparison studies between PBD and HC [34,38,39,43,44,45,46,47].

In the present study, we focused on WM regions showing the most significant differences in PBD compared to HC to explore differences between PBD and NPBD. First, TBSS analyses with threshold-free cluster enhancement (TFCE) were performed to explore the voxels showing changes in DTI measures between three groups (PBD, NPBD, and HC). Subsequently, ROI analyses were conducted by defining an ROI (TBSS-ROI) with the most significant connected voxels, showing the differences between DTI measures in PBD and HC in the TBSS results. In accordance with our hypothesis, TBSS-ROI exhibited severe WM abnormalities in PBD compared to NPBD. The present findings suggest the heterogeneity of WM abnormalities in BD in terms of psychotic symptoms, which provides important information on the differences and similarities between BD and SZ [46,49,51,52].

## 2. Materials and Methods

### 2.1. Subjects

The present study was approved by the Clinical Research Ethics Review Committee of Mie University Hospital. Each participant provided written informed consent after reading a description of this study. A total of 16 patients (8 males and 8 females) were recruited from inpatients and outpatients at Mie University Hospital. In total, 22 healthy controls (HC; 10 males and 12 females) were recruited from university office workers and their families, who had been educated for more than 12 years. The exclusion criteria for all subjects included a history of head trauma, neurological illness, serious medical or surgical illness, and substance use and abuse. None of the patients had any DSM-IV-TR axis I psychiatric comorbidities. A skilled neuroradiologist (M.M.) examined the MR images for signs of space-occupying lesions and significant cerebrovascular disease. High white matter signal intensities on T2-weighted images were seen in some asymptomatic individuals. All subjects with MR imaging showing infarct, focal parenchymal loss, and large patchy areas of white matter hyperintensities on their T2-weighted images were excluded. Patients were diagnosed as BD (8 PBD and 8 NPBD) based on the DSM-IV-TR. The mood state of the patients at the time of the scan was assessed via a clinical interview and a review of their medical charts. PBD was diagnosed based on the presence of lifetime psychosis defined by the current or past manifestation of hallucinations and/or delusions during manic or depressive episodes. In PBD and NPBD, 87.5% of the subjects were diagnosed as bipolar I and bipolar II, respectively. The healthy controls had no history of psychiatric illness, which was confirmed based on a brief interview modified from the non-patient edition of the Structured Clinical Interview for DSM-IV-TR axis I disorders (SCID-NP). Clinical interviews were performed by skilled psychiatrists (T.S. and H.T.), and all interviews for the healthy controls were performed by a skilled psychiatrist (T.S.). All clinical evaluations were reviewed and confirmed by consensus between skilled psychiatrists (T.S., E.M., H.T., and M.O.). The demographic data were compared between groups using Chi-square tests for nominal variables and Mann–Whitney and Kruskal–Wallis tests for continuous variables due to the small number of samples.

### 2.2. MRI and DTI Acquisitions

Diffusion tensor images and T1-weighted images were scanned using a 3.0T MRI scanner (Achieva, Philips Medical Systems, Best, The Netherlands) at Mie University Hospital. In the 3D T1-weighted images, sagittal acquisitions were comprised of 0.85 mm-thick slices with the following scan parameters: FOV = 250 × 250 mm; image matrix = 384 × 384; interslice gap = 0.85 mm; slice numbers = 220 slices; TE = 15.9 ms; TR = 300 ms; flip angle = 90°. Diffusion tensor images were acquired using a spin-echo echo-planar imaging sequence in 15 directions (b = 800 s/mm^2^), with one acquisition with no diffusion weighting (b = 0 s/mm^2^). The following scan parameters were used for the DTI:FOV = 240 × 240 mm; image matrix = 128 × 128; slice thickness = 2.5 mm with no interslice gap; slice numbers = 60 slices; TE = 70 ms; TR = 5400 ms; flip angle = 90°. Axial slices were aligned to the anterior commissure–posterior commissure (AC-PC) line in all subjects. Additional sequences (T2, FLAIR) were acquired to analyze and exclude the pathological findings. The imaging sequence parameters of 3D FLAIR were as follows: FOV = 250 mm; matrix = 256 × 184 (480 × 480 after reconstruction; in-plane resolution, 0.52 × 0.52 mm); section thickness = 1.14 mm with 0.57 mm overlap; no parallel imaging; TR/TE = 6000/shortest (approximately 400 ms); inversion time = 2000 ms; VFA (brain view FLAIR); TSE factor = 203; T2-prep = 125 ms (4 pluses); fat suppression with spectral presaturation inversion recovery, number of signals acquired = 2; scan time = 4 min and 54 s.

### 2.3. TBSS Analyses

We first performed whole-brain analyses of FA, MD, AD, and RD using TBSS [67], part of FSL ver. 6.0. Briefly, the diffusion-weighted images were corrected for eddy currents and head motion using eddy correction and then masked using BET. Images of FA were then calculated using DTIFIT (FMRIB’s Diffusion Toolbox 5.0). Images of MD, AD, and RD were also derived from eigenvalues obtained via the DTIFIT process; the images of MD and AD were directly obtained, and the images of RD, representing (the second eigenvalue + the third eigenvalue)/2, were computed using the command “fslmaths”. Tract-based spatial statistics were then performed. All FA images were non-linearly co-registered to the FA template (FMRIB58_FA_1 mm) and affine-aligned into the 1 × 1 × 1 mm MNI152 standard space. A mean FA image was created from all the subjects and thinned to create a mean skeletonized FA image using the threshold at the mean FA value of 0.2, which excluded gray matter and cerebral spinal fluid and exhibited the centers of white matter tracts common to the group. All subjects’ aligned FA data were then projected onto the mean skeletonized FA image. For non-linear registration, FNIRT in FSL ver. 6.0 was used, which enabled us to avoid the misalignment problems in the original version [68,69]. We also evaluated another registration algorithm, the Diffusion Tensor Imaging Tool Kit (DTI-TK) [70], which uses the full tensor information for the registration process [68]. The following voxel-wise statistical analyses on the FA skeleton voxels were then performed with the general linear model. These statistical inferences were performed with nonparametric permutation tests (5000 permutations) using TFCE within the FSL randomize program. The differences in mean FA between the three groups (PBD, NPBD, and HC) were examined. Age and gender were included as covariates. The results were corrected for multiple comparisons across voxels with family-wise error (FWE) corrections using TFCE, with a significance level of *p_tfce-FWE_* < 0.05. For the three-group comparison, the results of pairwise *t*-tests for exploring the direction of FA differences (6 contrasts) were corrected for multiple comparisons across contrasts by Bonferroni correction, with a significance level of *p_rice-FWE_* < 0.05/6 = 0.008. Group-by-age and group-by-gender interactions between the three groups were also analyzed, with a significance level of *p_tfce-FWE_* < 0.05/6 = 0.008. To investigate the group-by-age or group-by-gender interaction effects on FA, the differences in the age or gender slopes of FA between the three groups were investigated, with age and gender demeaned. Positive and negative aging effects in each group were separately analyzed, with a significance level of *p_tfce-FWE_* < 0.05/2 = 0.025 for two contrasts of positive and negative effects, while the gender effects in each group were also analyzed by assigning two indicator values for each gender. The other DTI measures (MD, AD, and RD) were also processed according to the guidance described in FSL Wiki (https://fsl.fmrib.ox.ac.uk/fsl/oldwiki/, accessed on 1 November 2020) and statistically examined in the same way as FA. The anatomical localization of significant voxels was identified based on white matter atlases [71,72,73] and the FSL-implemented JHU DTI-based white matter atlases.

### 2.4. TBSS-ROI Analyses

To obtain detailed information on the DTI measures of significantly connected voxels in the TBSS analyses, ROI analyses based on the significant results of TBSS (TBSS-ROI) instead of the arbitrary setting of ROI were performed, overcoming the limitations of conventional ROI methods. Using the command “cluster” from FSL utilities, an ROI mask comprised of the most significant connected voxels was created by thresholding the corrected *p*-value image in TBSS. The mean FA, MD, AD, and RD values of the ROI were then extracted from each subject’s FA, MD, AD, and RD skeleton, respectively, using the command “fslstats” from FSL utilities. The data were then analyzed using SPSS ver. 25 and JAMOVI ver. 2.3.28.0 software. The mean FA, MD, AD, and RD values of the ROI were compared between the three groups (PBD, NPBD, and HC) using a general linear model. Because of the reported association between WM microstructures and age [74] and gender [75], age and gender were included as covariates. Significant statistical values of TBSS-ROI analyses were set at *p* < 0.05 after Bonferroni correction for multiple comparisons. The small sample size in this study increases the chance of a type II error due to insufficient statistical power. In addition to *p*-values, the effect sizes and 95% confidence intervals for the differences in DTI measures between the three groups were evaluated.

## 3. Results

### 3.1. Subjects

The demographic and clinical profiles of the subjects are summarized in Table 1.

No significant differences were seen in age, gender, educational years, and handedness between PBD, NPBD, and HC, or age at onset and illness duration between PBD and NPBD.

### 3.2. TBSS Analyses

The group effects in the TBSS analyses are summarized in Table 2. The distribution of positive voxels was decided based on white matter atlases [71,72,73] and the FSL-implemented JHU DTI-based white matter atlases.

**Figure 1:**
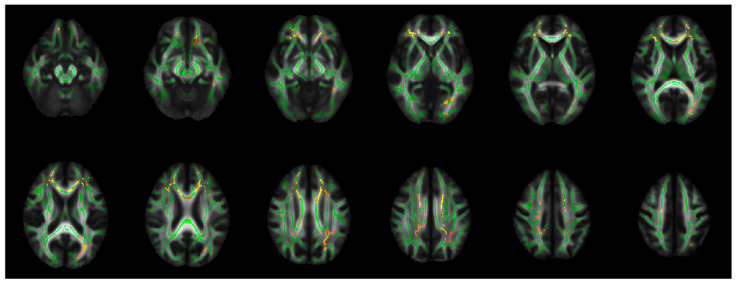
The regions of increased RD (red-yellow) in PBD compared to HC in the TBSS analyses, *p_tfce-FWE_* < 0.008, corrected for multiple comparisons across voxels and contrasts. Background: mean FA skeleton (green) and mean FA map. FA: fractional anisotropy; HC: healthy control; PBD: psychotic bipolar disorder; *p_tfce-FEW_*: corrected *p*-value for multiple comparisons across voxels by family-wise error (FWE) corrections using threshold-free cluster enhancement (TFCE); RD: radial diffusivity; TBSS: tract-based spatial statistics.

Increased RD in PBD compared to HC at *p_tfce-FEW_* < 0.008 was found in widespread WM regions, including bilateral frontal and parietal WM and left occipital and temporal WM, after corrections for multiple comparisons across voxels and contrasts (Table 2 and Figure 1).

Compared to NPBD, PBD exhibited a tendency of decreased FA and increased RD after corrections for multiple comparisons across voxels, which, however, did not survive after corrections for multiple comparisons across contrasts (Table 2).

Group-by-age interactions with gender included as a covariate were seen only between NPBD and HC on FA; the aging slope of FA was steeper in HC than NPBD in the left gCC, bCC, and ACR, extending to the superior frontal gyrus (SFG) WM at *p* < 0.008. Group-by-gender interactions with age included as a covariate were seen only between NPBD and HC on MD and RD; the gender slope of MD and RD were steeper in NPBD than HC in widespread WM at *p_tfce-FWE_* < 0.008.

The results from the alternative registration method using DTI-TK instead of the standard TBSS registration (FNIRT) confirmed the results from the FNIRT registration (Supplementary Materials Table S1). While the statistical significance was less than in the standard registration, increased RD in PBD compared to HC was seen in almost the same WM regions as in the FNIRT registration. The group-by-age and group-by-gender interactions in the FNIRT registration were also confirmed.

### 3.3. TBSS-ROI Analyses

**Figure 2:**
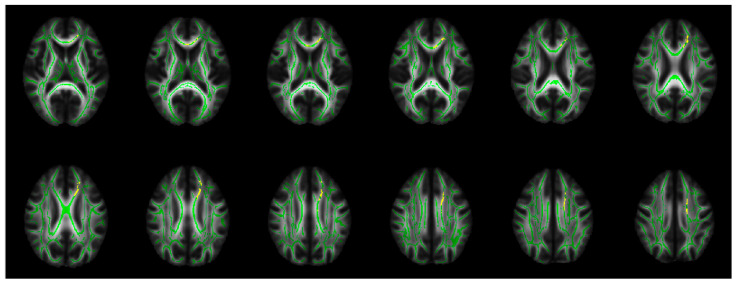
TBSS-ROI (yellow). The most significant 845 connected voxels showing increased RD in PBD compared to HC (*p_tfce-FWE_* < 0.005) in the TBSS analyses, overlaid on the mean FA skeleton (green) and mean FA map. FA: fractional anisotropy; HC: healthy control; PBD: psychotic bipolar disorder; *p_tfce-FEW_*: corrected *p*-value for multiple comparisons across voxels by family-wise error (FWE) corrections using threshold-free cluster enhancement (TFCE); RD: radial diffusivity; TBSS: tract-based spatial statistics.

Using the FSL command “cluster”, the most significant 845 connected voxels (*p_tfce-FWE_* < 0.005) were defined as TBSS-ROI from the corrected *p*-value image, showing significantly increased RD in PBD compared to HC. These voxels were distributed not in all WM tracts but in confined regions of the mean FA skeleton, corresponding to the bCC, left gCC, ACR, and SCR, extending to the SFG-WM (Figure 2).

All extracted mean FA, MD, AD, and RD values of TBSS-ROI in the three groups were first examined for normal distribution. The mean MD values of the ROI in PBD were not normally distributed and thus were excluded from the TBSS-ROI analyses. The results of the TBSS-ROI analyses are summarized in Table 3.

**Figure 3:**
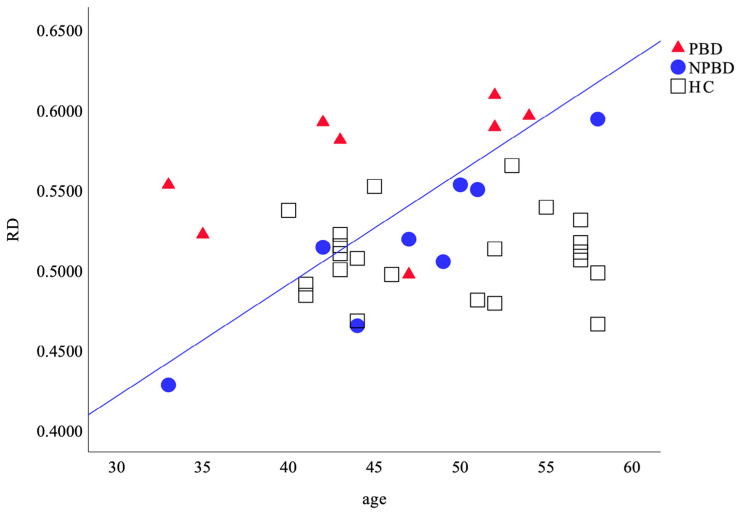
Scatterplots showing age-related radial diffusivity (RD) values of PBD, NPBD, and HC in TBSS-ROI. A significant positive aging slope was seen only in NPBD (solid blue line, y = 0.007x + 0.211). HC: healthy control (black square); NPBD: nonpsychotic bipolar disorder (blue circle); PBD: psychotic bipolar disorder (red triangle).

Group effects, including age and gender as covariates, demonstrated decreased FA and increased RD in PBD compared to NPBD and HC after Bonferroni corrections (Table 3). Because group-by-age interactions on FA and RD were significant between the three groups, the aging effects on FA and RD in the three groups were investigated separately. Age-related RD values of the three groups are plotted in Figure 3. Only NPBD showed a significant negative and positive linear aging slope estimate of FA (R^2^ = 0.925, *p* < 0.000) and RD (R^2^ = 0.822, *p* < 0.001), respectively. The increased RD compared to NPBD was consistent in PBD subjects, except for one early middle-aged one (Figure 3). The FA and RD values of PBD and NPBD were not correlated with either illness duration or age at onset.

Another TBSS-ROI analysis using DTI-TK instead of FNIRT was also performed. Using the FSL command “cluster”, 603 connected voxels (*p_tfce-FWE_* < 0.013) showing the tendency of increased RD in PBD compared to HC were defined as TBSS-ROI. TBSS-ROI in DTI-TK was smaller than FNIRT, but the results in FNIRT were confirmed (Supplementary Materials Table S2 and Figure S1).

## 4. Discussion

### 4.1. Comparison of WM Between PBD and NPBD

In the present study, the TBSS analyses exhibited increased RD in widespread WM tracts in PBD compared to HC (Table 2 and Figure 1). The most disrupted WM regions (TBSS-ROI) were distributed in confined regions in the bCC, left gCC, ACR, and SCR, extending to the SFG-WM (Figure 2). Decreased FA and increased RD in PBD compared to NPBD were detected in TBSS-ROI (Table 3).

No previous direct comparison studies of the WM microstructures between PBD and NPBD [29,40,61,62,63,64] have obtained significant findings in the gCC, ACR, and SCR. Values of FA [29,40,61,62,63,64] and MD [64], examined in previous studies, are frequently used but are nonspecific markers of WM changes [20], making it difficult to detect slight changes in specific factors of WM microstructures. In our TBSS-ROI analyses, the decreased FA in PBD compared to NPBD was not a robust finding because of the inconsistent distribution of trend voxels in the analyses. RD might be more suitable than FA for detecting WM changes [34]. In our study, the increased RD in PBD compared to NPBD in TBSS-ROI (Table 3 and Figure 3) was harmonized with the TBSS results, showing a tendency for increased RD in almost the same WM tracts (Table 2). While an increase in RD is influenced by increased extracellular water due to a variety of factors, such as axonal density, degree of myelination, axonal diameter, and inflammation [20,76], an increase in RD without AD changes suggests dysmyelination without axonal damage [77]. Taken together with previous studies using different techniques [78], suggesting that WM abnormalities in PBD are attributed not to axons but to myelin abnormalities, the increased RD in our results suggests severe dysmyelination in PBD compared to NPBD in confined WM regions in the bCC, left gCC, ACR, and SCR, extending to the SFG-WM. The present results did not confirm previous findings of WM differences between NPBD and HC [64], which could be due to the small sample size.

In the TBSS-ROI analyses, group-by-age interactions on RD between PBD, NPBD, and HC were observed, which suggests different age-associated trajectories of WM changes between the three middle-aged groups. Healthy WM tracts exhibit gradual age-associated RD increases after middle age due to aging [74]. The significant positive aging slope of RD in NPBD, in contrast to the non-significant aging slope in HC (Figure 3), is in accordance with a previous comparison of age-associated changes of the gCC between BD and HC in subjects of a wider age range [79]. Furthermore, the increased RD in PBD compared to NPBD was consistent, except for one early middle-aged subject, whereas the aging slope in PBD was not significant (Figure 3). Considering the hypothesis of delayed myelination and maturation in the anterior corpus callosum in pediatric PBD [80], differences in WM microstructures between PBD and NPBD might emerge before middle age. However, the present study employed a cross-sectional design in middle-aged subjects. The difference in age-associated WM changes in PBD and NPBD should be elucidated in future longitudinal studies investigating a wider age range. Illness duration and DTI measures were not correlated in our study in either PBD or NPBD, whereas negative correlations between illness duration and FA in the gCC and ACR [37] and the left cingulum [29] were observed in BD consortium studies. A group-by-gender interaction on RD between NPBD and HC was found in the TBSS analyses, which was not found in the TBSS-ROI analyses.

Disruption of the gCC [29,30,31,32,41,43,46,81,82,83], bCC [29,30,31,32,37,45,46,81,83], ACR, and SCR [28,29,46,83,84] has been repeatedly reported in BD [28,29,30,31,32,37,39,47,81,83,84] and PBD [43,45,46] compared to HC. A recent meta-analysis reported shared abnormalities between BD and SZ in the CC extending to the ACR and SCR [28]. Consortium studies [29,36] have established extensive shared WM abnormalities between BD and SZ compared to HC. Furthermore, the gCC and bCC showed strongly decreased FA effect sizes [29,36] in BD and SZ, and the ACR showed a strong effect in SZ [36]. These WM tracts are thus considered to be the most disrupted regions in extensive shared WM abnormalities in BD and SZ and are critically implicated in the pathophysiology of affective and psychotic symptoms in both illnesses. The present study demonstrated the heterogeneity of WM microstructures in BD in terms of the presence of psychotic symptoms in these WM tracts.

### 4.2. Functional Considerations

#### 4.2.1. Anterior Interhemispheric Communication

The gCC connects the left and right hemispheric prefrontal and orbitofrontal cortices, while the bCC connects both the hemispheric precentral and parietal regions [73,85]. The CC fibers maintain normal functional asymmetry by regulating each contralateral hemisphere [86,87]. Interhemispheric communication via the CC is essential for the coordination of sensory, motor, emotional, attention, decision-making, executive, visual, auditory, and language networks [85,86,87]. Emotional intelligence tests in subjects with agenesis of the CC have shown the impairment of strategic emotional reappraisal in social interactions with an intact emotional perception [88].

Combined studies of fMRI and DTI have reported the association between the FA value in the CC and the interhemispheric functional connectivity (FC) in the sensorimotor area [11], ventral PFC, and insula cortex [12], which are engaged in emotional processes [9,89]. Therefore, disruption of the CC induces emotional dysregulation in BD via the disfunction of interhemispheric communication between the sensorimotor area and the ventral PFC. There is a lack of fMRI data focused on the differences in interhemispheric FC between PBD and NPBD [48]. However, a previous DTI study showed decreased FA in the bCC in PBD compared to NPBD [61], which was supported by our results. Taking into account the correlation between FA in the CC and interhemispheric FC between brain regions implicated in emotional dysregulation in BD [11,12], more disrupted bCC in PBD compared to NPBD might contribute to severe mood symptoms in PBD compared to NPBD [42].

In terms of cognition, a combined study of fMRI and DTI [15] reported a correlation between the FA in the gCC and working memory and between FA in the bCC and processing speed. The present results show that greater alteration of the gCC in PBD compared to NPBD might contribute to more deteriorated working memory in PBD than NPBD [54].

Sustained attention in cognitive assessments is correlated with FA in the left bCC in BD [90], while psychomotor coordination is negatively associated with RD in the bCC in BD [4]. However, sustained attention and processing speed do not differ between PBD and NPBD [53], which contrasts with our expectation of severe dysmyelination in the bCC in PBD compared to NPBD.

#### 4.2.2. Cortico-Subcortical Pathways

The corona radiata (CR), a fan-shaped WM structure radiating into the cerebral hemisphere, consists of a mixture of various associations, projections, and callosal fibers [71,73,91]. Connecting fibers between cortex and subcortical structures, including the cortico-limbic [92], cortico-striatal [93], and thalamocortical inputs [94], pass through the CR, engaged in top-down and bottom-up modulation in these frontal cortical networks. Disruption of the CR can cause dysfunction of this modulation.

##### Fronto-Limbic Circuits

WM abnormalities in the fronto-limbic network [29,31,95,96,97,98] in BD have been established. In accordance with structural dysconnectivity, abruption of the FC of fronto-limbic circuits, including the anterior cingulate [99], amygdala [9,57], and hippocampus [100], in BD has been reported. Disturbed direct cognitive control for negative emotions in BD was indicated by the FC between the dorsolateral PFC and amygdala in emotion regulation tasks during fMRI [9]. Diminished FC between the medial PFC (mPFC) and the amygdala during emotional face tasks in BD has also been reported [16]. Amygdala hyperactivation has been found during the processing of fearful faces in BD [13]. In contrast, a paradoxically increased FC was found in the fronto-limbic circuits in the amygdala [57] and the hippocampal head [100], which could be due to a compensatory reaction to decreased structural connectivity. Reduced cortico-limbic inputs evoke stress-induced overactivation of the amygdala and dysregulation of the HPA axis, which play important roles in emotional dysregulation [89,92,98,101].

In terms of the differences between PBD and NPBD, previous fMRI studies have demonstrated a reduced global FC of the mPFC in PBD compared to NPBD [57]. In the present study, based on the distribution of TBSS-ROI (Figure 2), abundant connection fibers of the adjacent left medial frontal cortices, such as the mPFC and anterior cingulum, pass through TBSS-ROI. Dysmyelination in TBSS-ROI could cause structural dysconnectivity of the mPFC. The present results show severe abnormalities in TBSS-ROI in PBD compared to NPBD, in accordance with distinguished functional dysconnectivity of the mPFC between PBD and NPBD [57,58]. Greater reduced cortical inhibition from the mPFC in PBD compared to NPBD implies greater vulnerability of the amygdala to overactivation, which might be implicated in severe emotional dysregulation [1,51] and the emotional recognition deficits in PBD [102].

Furthermore, amygdala overactivation induces misattribution of the salience [93,103,104], which could be implicated in psychotic symptoms associated with emotional dysregulation [1,51] in PBD.

A previous DTI study reported increased FA in the left uncinate fasciculus, another inhibitory pathway to the amygdala, in PBD compared to NPBD [62], which could be a compensatory reaction to strengthen inhibitory control of the amygdala.

Additionally, the mPFC is the anterior node of the DMN, which is engaged in emotional processing and self-referential mental activity [18]. Dysfunctional DMN [105] and reduced connectivity in the mPFC in DMN in BD [106] have been reported. While self-referential processing in the midline cortical area is associated with vulnerability to psychosis [107], distinct FC in the mPFC in DMN between PBD and NPBD [58] has been reported. Another fMRI study reported less activation in the posterior cingulate cortex/precuneus, which is the hub of the DMN, in the evaluation of other related information in PBD compared to HC [6]. Taken together with these fMRI findings, the WM abnormalities in our TBSS-ROI indicate that structural dysconnectivity of the mPFC in PBD compared to NPBD might also contribute to simultaneous affective and psychotic symptoms in PBD due to the impaired processing of emotional and self-referential information.

Meanwhile, the FC between the mPFC and posterior cingulate cortex correlates with executive functioning in BD [108]. While attention, information processing, working memory, executive functions, and psychomotor coordination are associated with the integration of association fibers and the CR in BD [4], executive functioning in the Wisconsin Card Sorting Test in BD is negatively associated with RD in the left ACR [4]. Working memory tasks with or without emotional stimuli in BD showed opposite activation patterns in the region implicated in executive control [109]. The influence of emotional dysregulation on cognitive deficits is crucial in the pathophysiology of BD [3]. Given that severe structural dysconnectivity in the mPFC induces severe emotional dysregulation in PBD compared to NPBD, this might also contribute to severe neurocognitive dysfunction, including executive functioning [53,54] in PBD compared to NPBD.

In terms of social cognitive impairment in BD, less activation in the anterior cingulate during the ToM task in BD compared to HC suggests the implication of this region in an altered theory of mind [17]. Considering that our TBSS-ROI contained connecting fibers of the left anterior cingulate, severe dysmyelination of the fibers in TBSS-ROI in PBD related to NPBD might induce severe ToM impairment in PBD compared to NPBD, which is in harmony with the hypothesis that the severity of ToM deficits increases along the affective–psychotic spectrum [110].

Severe cognitive impairments are considered to affect severe clinical symptoms and poor psychosocial adaptation in PBD compared to NPBD [42,56].

##### Cortico-Striatal-Thalamic Loop

While the basal ganglia network is involved in the pathophysiology of mood disorders and psychosis [93], disconnections of the cortico-striatal [93] and thalamocortical [94] fibers passing through the CR provoke dysfunction in the cortico-striatal-thalamic loop.

An fMRI study of emotional stimuli demonstrated activation of the mediodorsal thalamic nucleus, a relay nucleus linking the basal ganglia and cortex, suggesting an important role for the mediodorsal thalamic nucleus in emotional processing, with coactivation of the striatum, cingulate, and anterior insula [14]. While abnormal resting-state FC in the frontal lobe and thalamus has been reported in early-onset BD [10], more severe functional dysconnectivity in the mediodorsal thalamic nucleus has been observed in PBD compared to NPBD [111]. Additionally, PBD and SZ share a reduction in thalamocortical connectivity [94].

Previous fMRI studies using emotional face tasks have reported fronto-limbic-striatal dysfunction in BD [13]. Functional dysconnectivity between the mPFC and putamen, along with elevated striatal dopamine synthesis capacity [112], is shared between PBD and SZ [104].

Reduced cortico-striatal WM integrity evokes an excess of striatal dopamine [93], which, in turn, induces the misattribution of salience [93,103,104], a mechanism implicated in psychotic symptoms. More severe disruption of the CR in PBD compared to NPBD may, therefore, contribute to emotional dysregulation and psychotic symptoms via dysconnectivity in the cortico-striatal-thalamic loop.

Altogether, the present study suggests severer impairments in anterior interhemispheric communication, the left fronto-limbic circuits, and cortico-striatal-thalamic loop in PBD compared to NPBD. These frontal disconnections are critical for dysfunctional emotion and cognition reappraisal, and the disturbance of control between cognitive and emotional process to each other [9,13,109] in BD, potentially explaining the simultaneous manifestation of affective and psychotic symptoms in PBD [42,56] via the pathological interaction between emotion and cognition [55,102,109,113].

### 4.3. Subtle Changes Within WM Tracts

Previous methodological approaches have limitations in detecting subtle changes within WM tracts.

In tract-specific analyses based on a priori hypotheses [29,61,62,63], including fiber tractography [29,61,62], the statistical effects of DTI measures such as FA, MD, AD, and RD in confined regions within WM tracts can be offset by unaffected regions when analyzing whole WM tracts. Whole-brain explorations without a priori hypotheses, such as TBSS [64] and voxel-based analysis [40,63], have additional limitations due to the conservative corrections for multiple comparisons and registration issues [68,69].

In the present study, we focused on WM regions showing the most significant differences in PBD compared to HC to explore differences between PBD and NPBD. A similar approach for generating an ROI has been used in previous secondary analyses following TBSS [82]. Unlike conventional or tract-specific ROIs, this irregularly shaped ROI spans multiple WM tracts on the FA skeleton produced by TBSS preprocessing. However, this method enabled us to obtain detailed information on the most significantly altered WM regions in PBD compared to HC.

In accordance with our hypothesis, our results distinguished WM microstructures between PBD and NPBD in the most disrupted WM regions in PBD compared to HC, which did not affect whole WM tracts but instead was confined within specific WM tracts. Concerning restricted changes, the value of DTI measures has been reported to vary along the WM tract [114,115]. Yeatman et al. [114] further described the possible localized pathological fiber changes within WM tracts. Taking into account the role of an individual oligodendrocyte for myelinating a particular set of axons [116], the region-specific deficit of oligodendrocytes in psychiatric diseases [35] might be implicated. Although the detailed functional topography of the fibers within WM tracts has not been elucidated, subtle dysmyelination has been demonstrated to cause cortical network dysfunction and behavioral impairment, such as catatonia-like symptoms in mutant mice [65]. While the implication of neuroinflammation in dysmyelination has been considered [31,76], a genetic mouse model [66] indicates that the microglia can be involved in subtle dysmyelination via oligodendrocyte dysfunction and the manifestation of behavioral impairment.

Although our data are preliminary, confined WM changes showing the differences between PBD and NPBD were found in WM tracts where strong effects of shared WM abnormalities between BD and SZ have been reported. In future investigations, topographical pathological changes within the CC and CR should be further explored in larger and controlled samples using more sophisticated methods from the perspective of oligodendrocyte dysfunction in BD.

### 4.4. Medication Effects

Medication effects on WM structure are controversial [23,117,118,119]. Antipsychotics [118] and mood stabilizers [96], including lithium [96,120] and antidepressants [96], reportedly could affect WM microstructures. Antidepressants affect the FA reduction in the left optic radiations [96]. Antipsychotics affect the FA reduction in parietal and occipital WM [118]. In contrast, lithium increases the FA in the hippocampal part of the cingulum [120], which suggests an improving effect of lithium on WM integrity. In the present study, most of the subjects, except 1 PBD and 3 NPBD subjects, were being treated with multiple medications. Antipsychotics were used more in PBD than in NPBD, while antidepressants and lamotrigine were used more in NPBD than in PBD (Table 1). Although our results are strengthened by previous studies showing minimal effects of medication [117], WM abnormalities in untreated first-episode PBD [47], and genetic liability in PBD [43], the potential effects of multiple medications on WM structure in the present study should be considered. Future studies should evaluate these results while adjusting for the effects of various medications as covariates in larger sample sizes.

### 4.5. Limitations

Some limitations should be noted when interpreting the present results. First, the small sample size and potential sampling bias may have affected the findings, in addition to reducing statistical power. These preliminary data on the confined abnormalities within these WM tracts should be replicated and validated in larger and controlled samples with more sophisticated methods of investigation. The diagnosis of bipolar I in PBD and NPBD was 87.5% and 12.5%, respectively. While no distinct WM abnormalities between bipolar I and II were found in a consortium study [29], some authors [121] have reported differing results. A recent systematic review reported that lifetime psychotic symptoms are two to three times higher in BDI than BDII [42]. Therefore, the differing inclusion ratios of BDI in PBD and NPBD are unlikely to have influenced the WM comparison results. Additionally, mood state-dependent WM alterations in BD should be considered. A gradient increase in WM abnormalities with cognitive impairment has been observed from the euthymic phase to the manic phase and, finally, the depressive phase [23]. While limbic and callosal WM abnormalities in euthymic BD patients, most of whom were PBD [122], support our results, different inclusion ratios of euthymic subjects in PBD and NPBD in the present study may have affected our results. Another limitation involves the DTI acquisition parameters, such as a low b-value and low directionality (15 directions) in this study, which could have reduced sensitivity to white matter microstructural abnormalities and contributed to the lack of significant differences between NPBD and HC. Methodological limitations of the imaging analyses should also be considered. TBSS is known to have issues with registration and skeletonizing processes, especially in WM tracts with crossing fibers, due to partial volume effects [68]. Caution should be applied in the assessment of TBSS results in the CC and CR because of the abundance of crossing fibers [68,69]. Additionally, TBSS inferences of non-FA measures, including MD, AD, and RD, rely on the FA skeleton, meaning the accuracy of the FA skeleton generation can affect the results of TBSS-ROI analyses. The low-directional image acquisition in the present study could have exaggerated this issue. To address misalignment problems, the FNIRT version (FSL 6.0) used in the present study enabled us to avoid the misalignment issues present in earlier versions. Furthermore, we confirmed the TBSS results from FNIRT using an alternative registration method with DTI-TK [70], which is a good solution to FNIRT’s limitations because it uses full tensor information [68].

## 5. Conclusions

Despite the limitation of the small sample size, the possible effects of medication, current mood state, and underpowered DTI acquisition parameters, the combined results of the TBSS and TBSS-ROI analyses indicate severe frontal WM disconnection in PBD compared to NPBD in the anterior interhemispheric communication and the left fronto-limbic circuits and cortico-striatal-thalamic loop, where strong effects of shared WM abnormalities between BD and SZ have been reported. These WM alterations might contribute to the pathophysiology of simultaneous manifestations of affective and psychotic symptoms in PBD, which are diagnostically located at the boundary between BD and SZ. In future investigation, topographic pathological changes within the CC and CR should be further explored in the perspective of the oligodendrocyte dysfunction in BD.

## Figures and Tables

**Table 1:** Subjects’ demographic and clinical profiles.

	PBD	NPBD	HC	*p* ^a^
Number of subjects	8	8	22	0.006 ^b^
Mean age (SD) (range)	44.75 (7.92) (33~54)	46.75 (7.36) (33~58)	49.09 (6.62) (40~58)	0.448
Gender (male/female)	4/4	4/4	10/12	1.000 ^b^
Educational years (SD) (range)	13.13 (3.04) (9~16)	14.88 (1.55) (12~17)	13.27 (1.58) (12~16)	0.115
Handedness (left/right) ^c^	1/7	2/6	1/21	0.192 ^b^
Bipolar I (n)/Bipolar II (n)	7/1	1/7		
Mood state (n)	D (3)/M (1)/E (4)	D (1)/E (7)		
Age at onset (SD) (range)	29.38 (7.63) (16~42)	29.63 (10.85) (15~51)		0.721
Duration of illness (SD) (range)	15.38 (8.26) (4~25)	17.25 (8.63) (7~32)		0.574
Auditory hallucinations (n)	6	-		
Visual hallucinations (n)	1	-		
Delusions (n)	7	-		
Disorganized thoughts (n)	8	-		
Medication				
Lithium (n)	4	4	N/A	1.000 ^b^
Valproic acid (n)	6	4	N/A	0.454 ^b^
Lamotrigine (n)	0	3	N/A	0.021 ^b^
Antidepressants (n)	1	5	N/A	0.006 ^b^
Antipsychotics (n)	5	1	N/A	0.006 ^b^
Benzodiazepines (n)	12	2	N/A	0.151 ^b^
Other (n)	3	2	N/A	0.210 ^b^

HC: healthy control; NPBD: nonpsychotic bipolar disorder; PBD: psychotic bipolar disorder; SD: standard deviation; D: depressive state; M: manic state; E: euthymic state in mood state. ^a^ *p*-value for comparing analyses (PBD vs. NPBD vs. HC in the number of subjects, mean age, gender, educational years, and handedness; PBD vs. NPBD in age at onset, duration of illness, and medication). ^b^ Chi-squared test *p*-value, Fisher’s exact test. ^c^ Handedness is based on self-report. Antidepressants included escitalopram (1 PBD and 1 NPBD), paroxetine (1 NPBD), amitriptyline (1 NPBD), fluvoxamine (1 NPBD), and sulpiride (1 NPBD). Antipsychotics included quetiapine (1 PBD), perphenazine (1 PBD), levomepromazine (1 PBD), risperidone (1PBD), and olanzapine (1 PBD and 1 NPBD). Benzodiazepines included ethyl loflazepate (3 PBD), alprazolam (1 PBD), bromazepam (1 PBD), flunitrazepam (3 PBD), brotizolam (3 PBD and 1 NPBD), quazepam (1 NPBD), and rilmazafone (1 PBD). Other medications included zolpidem (1 NPBD), levothyroxine (1 PBD and 1 NPBD), Tsumura-Kampo Tokisyakuyakusanryo (1 PBD), and trihexyphenidyl hydrochloride (1 PBD). Both PBD and NPBD subjects were under multiple medication treatments except 1 PBD (valproic acid) and 3 NPBD subjects (valproic acid, lithium, and lamotrigine), who were treated with a single medication.

**Table 2:** Group effects in the TBSS analyses.

PBD Compared to HC	
Findings	WM Tracts
Decreased FA (*p_tfce-FWE_* < 0.012 *)	Left parietal and occipital WM
Increased MD (*p_tfce-FWE_* < 0.012 *)	Left Fmin, gCC, bCC, ACR, SCR, and inferior frontal WM
Increased RD (*p_tfce-FWE_* < 0.008 **)	Bilateral orbitofrontal cortex WM, Fmin, gCC, bCC, ACR, SCR, PCR, anterior cingulum, inferior frontal WM, bottom part of the superior parietal lobule WM, posterior part of the superior longitudinal fasciculus, and posterior cingulum The left posterior part of the inferior fronto-occipital fasciculus and ILF, posterior thalamic radiation, optic radiation, lingual gyrus WM, splenium of the CC, forceps major, and the temporal part of the IFL
**PBD Compared to NPBD**	
**Findings**	**WM Tracts**
Decreased FA (*p_tfce-FWE_* < 0.014 *)	Left parietal and occipital WM
Increased RD (*p_tfce-FWE_* < 0.014 *)	Left Fmin, gCC, bCC, and ACR

Age and gender were included as covariates. * Corrected for multiple comparisons across voxels. ** Corrected for multiple comparisons across voxels and contrasts. ACR: anterior corona radiata; bCC: body of corpus callosum; FA: fractional anisotropy; Fmin: forceps minor; gCC: genu of the corpus callosum; ILF: inferior longitudinal fasciculus; MD: mean diffusivity; NPBD: nonpsychotic bipolar disorder; PBD: psychotic bipolar disorder; PCR: posterior corona radiata; *p_tfce-FEW_*: corrected *p*-value for multiple comparisons across voxels by family-wise error (FWE) corrections using threshold-free cluster enhancement (TFCE); RD: radial diffusivity; SCR: superior corona radiata; WM: white matter.

**Table 3:** Results of the TBSS-ROI analyses.

DTI Measure	PBD	NPBD	HC	3 Groups ANOVA (*F*)	3 Groups ANOVA (*p*) Effect Size (*η*^2^)	3 Groups Group × Age (*p*) Effect Size (*η*^2^)	3 Groups Group × Gender (*p*) Effect Size (*η*^2^)	PBD vs. NPBD (*p*) Effect Size (95% CI)	PBD vs. HC (*p*) Effect Size (95% CI)	NPBD vs. HC (*p*) Effect Size (95% CI)
	Mean (SD)	Mean (SD)	Mean (SD)							
FA	0.501 (0.039)	0.542 (0.050)	0.547 (0.026)	7.840	0.002 * 0.288	0.002 * 0.312	0.618 0.040	0.029 * d = −1.383 [−0.087, −0.004]	0.001 * d = −1.697 [−0.092, 0.020]	1.000 *d* = −0.314 [−0.045, 0.024]
AD	1.358 (0.038)	1.348 (0.058)	1.352 (0.047)	0.059	0.942 0.035	0.210 0.085	0.179 0.089	1.000 *d* = 0.167 [−0.054, 0.070]	1.000 *d* = 0.0542 [−0.051, 0.056]	1.000 *d* = −0.1125 [−0.057, 0.046]
RD	0.567 (0.040)	0.516 (0.052)	0.509 (0.026)	11.960	0.000 * 0.380	0.003 * 0.348	0.134 0.134	0.006 * d = 1.706 [0.014, 0.098]	0.000 * d = 2.096 [0.033, 0.105]	1.000 *d* = 0.3895 [−0.022, 0.048]

FA: fractional anisotropy; AD: axial diffusivity; RD: radial diffusivity. 95% CI: 95% confidence interval for the difference between DTI measures. Age and gender were included as covariates. * Bonferroni corrected.

## Data Availability

The data reported in this article are available from the corresponding author upon adequate request due to privacy.

## Supplementary Materials

The following supporting information can be downloaded at https://www.mdpi.com/article/10.3390/brainsci15020108/s1: Figure S1: Scatterplots of RD values in TBSS-ROI based on registration using DTI-TK; Table S1: Group effects in the TBSS analyses based on registration using DTI-TK; Table S2: Results of the TBSS-ROI analyses based on registration using DTI-TK.

## Abbreviations

The following abbreviations are used in this manuscript:

ACR	anterior corona radiata
AD	axial diffusivity
bCC	body of the corpus callosum
BD	bipolar disorder
CC	corpus callosum
DMN	default mode network
DTI	diffusion tensor imaging
DTI-TK	Diffusion Tensor Imaging Tool Kit
FA	fractional anisotropy
FC	functional connectivity
Fmin	forceps minor
fMRI	functional MRI
FEW	Family-wise error
gCC	genu of the corpus callosum
HC	healthy controls
ILF	inferior longitudinal fasciculus
MD	mean diffusivity
mPFC	medial prefrontal cortex
NPBD	nonpsychotic bipolar disorder
PBD	psychotic bipolar disorder
PCR	posterior corona radiata
PFC	prefrontal cortex
RD	radial diffusivity
SCR	superior corona radiata
SD	standard deviation
SFG	superior frontal gyrus
SZ	schizophrenia
TBSS	tract-based spatial statistics
TFCE	threshold-free cluster enhancement
ToM	theory of mind
WM	white matter

## References

[1] American Psychiatric Association (2000). Diagnostic and Statistical Manual of Mental Disorders, Fourth Edition, Text Revision (DSMIV-TR).

[2] Oliva V., De Prisco M., Fico G., Possidente C., Bort M., Fortea L., Montejo L., Anmella G., Hidalgo-Mazzei D., Murru A. (2024). Highest correlations between emotion regulation strategies and mood symptoms in bipolar disorder: A systematic review and Bayesian network meta-analysis. Neurosci. Biobehav. Rev..

[3] Lima I.M.M., Peckham A.D., Johnson S.L. (2018). Cognitive deficits in bipolar disorders: Implications for emotion. Clin. Psychol. Rev..

[4] Poletti S., Bollettini I., Mazza E., Locatelli C., Radaelli D., Vai B., Smeraldi E., Colombo C., Benedetti F. (2015). Cognitive performances associate with measures of white matter integrity in bipolar disorder. J. Affect. Disord..

[5] Mesbah R., Koenders M.A., van der Wee N.J.A., Giltay E.J., van Hemert A.M., de Leeuw M. (2023). Association Between the Fronto-Limbic Network and Cognitive and Emotional Functioning in Individuals With Bipolar Disorder: A Systematic Review and Meta-analysis. JAMA Psychiatry.

[6] Zhang L., Opmeer E.M., Ruhe H.G., Aleman A., van der Meer L. (2015). Brain activation during self- and other-reflection in bipolar disorder with a history of psychosis: Comparison to schizophrenia. NeuroImage Clin..

[7] Schumer M.C., Chase H.W., Rozovsky R., Eickhoff S.B., Phillips M.L. (2023). Prefrontal, parietal, and limbic condition-dependent differences in bipolar disorder: A large-scale meta-analysis of functional neuroimaging studies. Mol. Psychiatry.

[8] Jimenez A.M., Riedel P., Lee J., Reavis E.A., Green M.F. (2019). Linking resting-state networks and social cognition in schizophrenia and bipolar disorder. Hum. Brain Mapp..

[9] Zhang L., Opmeer E.M., van der Meer L., Aleman A., Ćurčić-Blake B., Ruhé H.G. (2018). Altered frontal-amygdala effective connectivity during effortful emotion regulation in bipolar disorder. Bipolar Disord..

[10] Cattarinussi G., Bellani M., Maggioni E., Sambataro F., Brambilla P., Delvecchio G. (2022). Resting-state functional connectivity and spontaneous brain activity in early-onset bipolar disorder: A review of functional Magnetic Resonance Imaging studies. J. Affect. Disord..

[11] Ishida T., Donishi T., Iwatani J., Yamada S., Takahashi S., Ukai S., Shinosaki K., Terada M., Kaneoke Y. (2017). Interhemispheric disconnectivity in the sensorimotor network in bipolar disorder revealed by functional connectivity and diffusion tensor imaging analysis. Heliyon.

[12] Yasuno F., Kudo T., Matsuoka K., Yamamoto A., Takahashi M., Nakagawara J., Nagatsuka K., Iida H., Kishimoto T. (2016). Interhemispheric functional disconnection because of abnormal corpus callosum integrity in bipolar disorder type II. BJPsych Open.

[13] Brotman M.A., Tseng W.L., Olsavsky A.K., Fromm S.J., Muhrer E.J., Rutenberg J.G., Deveney C.M., Adleman N.E., Zarate C.A., Pine D.S. (2014). Fronto-limbic-striatal dysfunction in pediatric and adult patients with bipolar disorder: Impact of face emotion and attentional demands. Psychol. Med..

[14] Metzger C.D., Eckert U., Steiner J., Sartorius A., Buchmann J.E., Stadler J., Tempelmann C., Speck O., Bogerts B., Abler B. (2010). High field FMRI reveals thalamocortical integration of segregated cognitive and emotional processing in mediodorsal and intralaminar thalamic nuclei. Front. Neuroanat..

[15] McKenna B.S., Theilmann R.J., Sutherland A.N., Eyler L.T. (2015). Fusing Functional MRI and Diffusion Tensor Imaging Measures of Brain Function and Structure to Predict Working Memory and Processing Speed Performance among Inter-episode Bipolar Patients. J. Int. Neuropsychol. Soc. JINS.

[16] Tseng W.L., Thomas L.A., Harkins E., Stoddard J., Zarate C.A., Pine D.S., Leibenluft E., Brotman M.A. (2016). Functional connectivity during masked and unmasked face emotion processing in bipolar disorder. Psychiatry Res. Neuroimaging.

[17] Malhi G.S., Lagopoulos J., Das P., Moss K., Berk M., Coulston C.M. (2008). A functional MRI study of Theory of Mind in euthymic bipolar disorder patients. Bipolar Disord..

[18] Raichle M.E. (2015). The brain’s default mode network. Annu. Rev. Neurosci..

[19] Beaulieu C., Johansen-Berg H., Behrens T.E. (2014). The Biological Basis Of Diffusion Anisotropy. Diffusion MRI: From Quantitative Measurement to In Vivo Neuroanatomy.

[20] Alexander A.L., Lee J.E., Lazar M., Field A.S. (2007). Diffusion tensor imaging of the brain. Neurother. J. Am. Soc. Exp. NeuroTher..

[21] Peters B.D., Ikuta T., DeRosse P., John M., Burdick K.E., Gruner P., Prendergast D.M., Szeszko P.R., Malhotra A.K. (2014). Age-related differences in white matter tract microstructure are associated with cognitive performance from childhood to adulthood. Biol. Psychiatry.

[22] Bauer I.E., Ouyang A., Mwangi B., Sanches M., Zunta-Soares G.B., Keefe R.S., Huang H., Soares J.C. (2015). Reduced white matter integrity and verbal fluency impairment in young adults with bipolar disorder: A diffusion tensor imaging study. J. Psychiatr. Res..

[23] Magioncalda P., Martino M., Conio B., Piaggio N., Teodorescu R., Escelsior A., Marozzi V., Rocchi G., Roccatagliata L., Northoff G. (2016). Patterns of microstructural white matter abnormalities and their impact on cognitive dysfunction in the various phases of type I bipolar disorder. J. Affect. Disord..

[24] Linke J.O., Stavish C., Adleman N.E., Sarlls J., Towbin K.E., Leibenluft E., Brotman M.A. (2020). White matter microstructure in youth with and at risk for bipolar disorder. Bipolar Disord..

[25] Lima Santos J.P., Bertocci M., Bebko G., Goldstein T., Kim T., Iyengar S., Bonar L., Gill M., Merranko J., Yendiki A. (2022). White Matter Correlates of Early-Onset Bipolar Illness and Predictors of One-Year Recurrence of Depression in Adults with Bipolar Disorder. J. Clin. Med..

[26] Sarubbo S., De Benedictis A., Merler S., Mandonnet E., Balbi S., Granieri E., Duffau H. (2015). Towards a functional atlas of human white matter. Hum. Brain Mapp..

[27] Marstaller L., Williams M., Rich A., Savage G., Burianová H. (2015). Aging and large-scale functional networks: White matter integrity, gray matter volume, and functional connectivity in the resting state. Neuroscience.

[28] Zhao G., Lau W.K.W., Wang C., Yan H., Zhang C., Lin K., Qiu S., Huang R., Zhang R. (2022). A Comparative Multimodal Meta-analysis of Anisotropy and Volume Abnormalities in White Matter in People Suffering From Bipolar Disorder or Schizophrenia. Schizophr. Bull..

[29] Favre P., Pauling M., Stout J., Hozer F., Sarrazin S., Abe C., Alda M., Alloza C., Alonso-Lana S., Andreassen O.A. (2019). Widespread white matter microstructural abnormalities in bipolar disorder: Evidence from mega- and meta-analyses across 3033 individuals. Neuropsychopharmacol. Off. Publ. Am. Coll. Neuropsychopharmacol..

[30] Benedetti F., Bollettini I. (2014). Recent findings on the role of white matter pathology in bipolar disorder. Harv. Rev. Psychiatry.

[31] Brambilla P., Bellani M., Yeh P.H., Soares J.C., Tansella M. (2009). White matter connectivity in bipolar disorder. Int. Rev. Psychiatry.

[32] Mahon K., Burdick K.E., Szeszko P.R. (2010). A role for white matter abnormalities in the pathophysiology of bipolar disorder. Neurosci. Biobehav. Rev..

[33] Ji E., Lejuste F., Sarrazin S., Houenou J. (2019). From the microscope to the magnet: Disconnection in schizophrenia and bipolar disorder. Neurosci. Biobehav. Rev..

[34] Nenadic I., Hoof A., Dietzek M., Langbein K., Reichenbach J.R., Sauer H., Gullmar D. (2017). Diffusion tensor imaging of cingulum bundle and corpus callosum in schizophrenia vs. bipolar disorder. Psychiatry Res. Neuroimaging.

[35] Valdés-Tovar M., Rodríguez-Ramírez A.M., Rodríguez-Cárdenas L., Sotelo-Ramírez C.E., Camarena B., Sanabrais-Jiménez M.A., Solís-Chagoyán H., Argueta J., López-Riquelme G.O. (2022). Insights into myelin dysfunction in schizophrenia and bipolar disorder. World J. Psychiatry.

[36] Kelly S., Jahanshad N., Zalesky A., Kochunov P., Agartz I., Alloza C., Andreassen O.A., Arango C., Banaj N., Bouix S. (2017). Widespread white matter microstructural differences in schizophrenia across 4322 individuals: Results from the ENIGMA Schizophrenia DTI Working Group. Mol. Psychiatry.

[37] Koshiyama D., Fukunaga M., Okada N., Morita K., Nemoto K., Usui K., Yamamori H., Yasuda Y., Fujimoto M., Kudo N. (2020). White matter microstructural alterations across four major psychiatric disorders: Mega-analysis study in 2937 individuals. Mol. Psychiatry.

[38] Cui L., Chen Z., Deng W., Huang X., Li M., Ma X., Huang C., Jiang L., Wang Y., Wang Q. (2011). Assessment of white matter abnormalities in paranoid schizophrenia and bipolar mania patients. Psychiatry Res..

[39] Ho N.F., Li Z., Ji F., Wang M., Kuswanto C.N., Sum M.Y., Tng H.Y., Sitoh Y.Y., Sim K., Zhou J. (2017). Hemispheric lateralization abnormalities of the white matter microstructure in patients with schizophrenia and bipolar disorder. J. Psychiatry Neurosci. JPN.

[40] Sussmann J.E., Lymer G.K., McKirdy J., Moorhead T.W., Munoz Maniega S., Job D., Hall J., Bastin M.E., Johnstone E.C., Lawrie S.M. (2009). White matter abnormalities in bipolar disorder and schizophrenia detected using diffusion tensor magnetic resonance imaging. Bipolar Disord..

[41] Li J., Kale Edmiston E., Chen K., Tang Y., Ouyang X., Jiang Y., Fan G., Ren L., Liu J., Zhou Y. (2014). A comparative diffusion tensor imaging study of corpus callosum subregion integrity in bipolar disorder and schizophrenia. Psychiatry Res..

[42] Chakrabarti S., Singh N. (2022). Psychotic symptoms in bipolar disorder and their impact on the illness: A systematic review. World J. Psychiatry.

[43] Chaddock C.A., Barker G.J., Marshall N., Schulze K., Hall M.H., Fern A., Walshe M., Bramon E., Chitnis X.A., Murray R. (2009). White matter microstructural impairments and genetic liability to familial bipolar I disorder. Br. J. Psychiatry J. Ment. Sci..

[44] Emsell L., Chaddock C., Forde N., Van Hecke W., Barker G.J., Leemans A., Sunaert S., Walshe M., Bramon E., Cannon D. (2013). White matter microstructural abnormalities in families multiply affected with bipolar I disorder: A diffusion tensor tractography study. Psychol. Med..

[45] Kumar J., Iwabuchi S., Oowise S., Balain V., Palaniyappan L., Liddle P.F. (2015). Shared white-matter dysconnectivity in schizophrenia and bipolar disorder with psychosis. Psychol. Med..

[46] Skudlarski P., Schretlen D.J., Thaker G.K., Stevens M.C., Keshavan M.S., Sweeney J.A., Tamminga C.A., Clementz B.A., O’Neil K., Pearlson G.D. (2013). Diffusion tensor imaging white matter endophenotypes in patients with schizophrenia or psychotic bipolar disorder and their relatives. Am. J. Psychiatry.

[47] Lu L.H., Zhou X.J., Keedy S.K., Reilly J.L., Sweeney J.A. (2011). White matter microstructure in untreated first episode bipolar disorder with psychosis: Comparison with schizophrenia. Bipolar Disord..

[48] Hozer F., Houenou J. (2016). Can neuroimaging disentangle bipolar disorder?. J. Affect. Disord..

[49] Ivleva E.I., Morris D.W., Moates A.F., Suppes T., Thaker G.K., Tamminga C.A. (2010). Genetics and intermediate phenotypes of the schizophrenia--bipolar disorder boundary. Neurosci. Biobehav. Rev..

[50] Hatotani N. (1996). The concept of ‘atypical psychoses’: Special reference to its development in Japan. Psychiatry Clin. Neurosci..

[51] Möller H.J. (2008). Systematic of psychiatric disorders between categorical and dimensional approaches: Kraepelin’s dichotomy and beyond. Eur. Arch. Psychiatry Clin. Neurosci..

[52] Grunze H., Cetkovich-Bakmas M. (2021). “Apples and pears are similar, but still different things.” Bipolar disorder and schizophrenia- discrete disorders or just dimensions?. J. Affect. Disord..

[53] Glahn D.C., Bearden C.E., Barguil M., Barrett J., Reichenberg A., Bowden C.L., Soares J.C., Velligan D.I. (2007). The neurocognitive signature of psychotic bipolar disorder. Biol. Psychiatry.

[54] Bora E. (2018). Neurocognitive features in clinical subgroups of bipolar disorder: A meta-analysis. J. Affect. Disord..

[55] Thaler N.S., Strauss G.P., Sutton G.P., Vertinski M., Ringdahl E.N., Snyder J.S., Allen D.N. (2013). Emotion perception abnormalities across sensory modalities in bipolar disorder with psychotic features and schizophrenia. Schizophr. Res..

[56] Marneros A., Rottig S., Rottig D., Tscharntke A., Brieger P. (2009). Bipolar I disorder with mood-incongruent psychotic symptoms: A comparative longitudinal study. Eur. Arch. Psychiatry Clin. Neurosci..

[57] Anticevic A., Brumbaugh M.S., Winkler A.M., Lombardo L.E., Barrett J., Corlett P.R., Kober H., Gruber J., Repovs G., Cole M.W. (2013). Global prefrontal and fronto-amygdala dysconnectivity in bipolar I disorder with psychosis history. Biol. Psychiatry.

[58] Zhong Y., Wang C., Gao W., Xiao Q., Lu D., Jiao Q., Su L., Lu G. (2019). Aberrant Resting-State Functional Connectivity in the Default Mode Network in Pediatric Bipolar Disorder Patients with and without Psychotic Symptoms. Neurosci. Bull..

[59] Hibar D.P., Westlye L.T., Doan N.T., Jahanshad N., Cheung J.W., Ching C.R.K., Versace A., Bilderbeck A.C., Uhlmann A., Mwangi B. (2017). Cortical abnormalities in bipolar disorder: An MRI analysis of 6503 individuals from the ENIGMA Bipolar Disorder Working Group. Mol. Psychiatry.

[60] Sarrazin S., d’Albis M.A., McDonald C., Linke J., Wessa M., Phillips M., Delavest M., Emsell L., Versace A., Almeida J. (2015). Corpus callosum area in patients with bipolar disorder with and without psychotic features: An international multicentre study. J. Psychiatry Neurosci. JPN.

[61] Sarrazin S., Poupon C., Linke J., Wessa M., Phillips M., Delavest M., Versace A., Almeida J., Guevara P., Duclap D. (2014). A multicenter tractography study of deep white matter tracts in bipolar I disorder: Psychotic features and interhemispheric disconnectivity. JAMA Psychiatry.

[62] Ji A., Godwin D., Rutlin J., Kandala S., Shimony J.S., Mamah D. (2017). Tract-based analysis of white matter integrity in psychotic and nonpsychotic bipolar disorder. J. Affect. Disord..

[63] Brown J.A., Jackson B.S., Burton C.R., Hoy J.E., Sweeney J.A., Pearlson G.D., Keshavan M.S., Keedy S.S., Gershon E.S., Tamminga C.A. (2021). Reduced white matter microstructure in bipolar disorder with and without psychosis. Bipolar Disord..

[64] Lee D.K., Lee H., Ryu V., Kim S.W., Ryu S. (2022). Different patterns of white matter microstructural alterations between psychotic and non-psychotic bipolar disorder. PLoS ONE.

[65] Poggi G., Boretius S., Mobius W., Moschny N., Baudewig J., Ruhwedel T., Hassouna I., Wieser G.L., Werner H.B., Goebbels S. (2016). Cortical network dysfunction caused by a subtle defect of myelination. Glia.

[66] Arinrad S., Depp C., Siems S.B., Sasmita A.O., Eichel M.A., Ronnenberg A., Hammerschmidt K., Luders K.A., Werner H.B., Ehrenreich H. (2023). Isolated catatonia-like executive dysfunction in mice with forebrain-specific loss of myelin integrity. eLife.

[67] Smith S.M., Jenkinson M., Johansen-Berg H., Rueckert D., Nichols T.E., Mackay C.E., Watkins K.E., Ciccarelli O., Cader M.Z., Matthews P.M. (2006). Tract-based spatial statistics: Voxelwise analysis of multi-subject diffusion data. NeuroImage.

[68] Bach M., Laun F.B., Leemans A., Tax C.M., Biessels G.J., Stieltjes B., Maier-Hein K.H. (2014). Methodological considerations on tract-based spatial statistics (TBSS). NeuroImage.

[69] Jbabdi S., Behrens T.E., Smith S.M. (2010). Crossing fibres in tract-based spatial statistics. NeuroImage.

[70] Zhang H., Yushkevich P.A., Alexander D.C., Gee J.C. (2006). Deformable registration of diffusion tensor MR images with explicit orientation optimization. Med. Image Anal..

[71] Wakana S., Jiang H., Nagae-Poetscher L.M., Van Zijl P.C., Mori S. (2004). Fiber tract-based atlas of human white matter anatomy. Radiology.

[72] Oishi K., Faria A.V., van Zijl P.C., Mori S. (2010). MRI Atlas of Human White Matter.

[73] Catani M., Thiebaut de Schotten M. (2008). A diffusion tensor imaging tractography atlas for virtual in vivo dissections. Cortex.

[74] Westlye L.T., Walhovd K.B., Dale A.M., Bjornerud A., Due-Tonnessen P., Engvig A., Grydeland H., Tamnes C.K., Ostby Y., Fjell A.M. (2010). Life-span changes of the human brain white matter: Diffusion tensor imaging (DTI) and volumetry. Cereb. Cortex.

[75] Kanaan R.A., Chaddock C., Allin M., Picchioni M.M., Daly E., Shergill S.S., McGuire P.K. (2014). Gender influence on white matter microstructure: A tract-based spatial statistics analysis. PLoS ONE.

[76] Benedetti F., Poletti S., Hoogenboezem T.A., Mazza E., Ambrée O., de Wit H., Wijkhuijs A.J., Locatelli C., Bollettini I., Colombo C. (2016). Inflammatory cytokines influence measures of white matter integrity in Bipolar Disorder. J. Affect. Disord..

[77] Song S.-K., Sun S.-W., Ramsbottom M.J., Chang C., Russell J., Cross A.H. (2002). Dysmyelination Revealed through MRI as Increased Radial (but Unchanged Axial) Diffusion of Water. NeuroImage.

[78] Lewandowski K.E., Ongur D., Sperry S.H., Cohen B.M., Sehovic S., Goldbach J.R., Du F. (2015). Myelin vs Axon Abnormalities in White Matter in Bipolar Disorder. Neuropsychopharmacol. Off. Publ. Am. Coll. Neuropsychopharmacol..

[79] Toteja N., Guvenek-Cokol P., Ikuta T., Kafantaris V., Peters B.D., Burdick K.E., John M., Malhotra A.K., Szeszko P.R. (2015). Age-associated alterations in corpus callosum white matter integrity in bipolar disorder assessed using probabilistic tractography. Bipolar Disord..

[80] James A., Hough M., James S., Burge L., Winmill L., Nijhawan S., Matthews P.M., Zarei M. (2011). Structural brain and neuropsychometric changes associated with pediatric bipolar disorder with psychosis. Bipolar Disord..

[81] Videtta G., Squarcina L., Rossetti M.G., Brambilla P., Delvecchio G., Bellani M. (2023). White matter modifications of corpus callosum in bipolar disorder: A DTI tractography review. J. Affect. Disord..

[82] Barnea-Goraly N., Chang K.D., Karchemskiy A., Howe M.E., Reiss A.L. (2009). Limbic and corpus callosum aberrations in adolescents with bipolar disorder: A tract-based spatial statistics analysis. Biol. Psychiatry.

[83] Lagopoulos J., Hermens D.F., Hatton S.N., Tobias-Webb J., Griffiths K., Naismith S.L., Scott E.M., Hickie I.B. (2013). Microstructural white matter changes in the corpus callosum of young people with Bipolar Disorder: A diffusion tensor imaging study. PLoS ONE.

[84] Pavuluri M.N., Yang S., Kamineni K., Passarotti A.M., Srinivasan G., Harral E.M., Sweeney J.A., Zhou X.J. (2009). Diffusion tensor imaging study of white matter fiber tracts in pediatric bipolar disorder and attention-deficit/hyperactivity disorder. Biol. Psychiatry.

[85] Fabri M., Pierpaoli C., Barbaresi P., Polonara G. (2014). Functional topography of the corpus callosum investigated by DTI and fMRI. World J. Radiol..

[86] van der Knaap L.J., van der Ham I.J. (2011). How does the corpus callosum mediate interhemispheric transfer? A review. Behav. Brain Res..

[87] Schutter D.J., Harmon-Jones E. (2013). The corpus callosum: A commissural road to anger and aggression. Neurosci. Biobehav. Rev..

[88] Anderson L.B., Paul L.K., Brown W.S. (2017). Emotional Intelligence in Agenesis of the Corpus Callosum. Arch. Clin. Neuropsychol. Off. J. Natl. Acad. Neuropsychol..

[89] Strakowski S.M., Adler C.M., Almeida J., Altshuler L.L., Blumberg H.P., Chang K.K.D., DelBello M.P., Frangou S., McIntosh A., Phillips M.L. (2012). The functional neuroanatomy of bipolar disorder: A consensus model. Bipolar Disord..

[90] Masuda Y., Okada G., Takamura M., Shibasaki C., Yoshino A., Yokoyama S., Ichikawa N., Okuhata S., Kobayashi T., Yamawaki S. (2020). White matter abnormalities and cognitive function in euthymic patients with bipolar disorder and major depressive disorder. Brain Behav..

[91] Yakar F., Eroglu U., Peker E., Armagan E., Comert A., Ugur H.C. (2018). Structure of corona radiata and tapetum fibers in ventricular surgery. J. Clin. Neurosci. Off. J. Neurosurg. Soc. Australas..

[92] Price J.L., Drevets W.C. (2012). Neural circuits underlying the pathophysiology of mood disorders. Trends Cogn. Sci..

[93] Macpherson T., Hikida T. (2019). Role of basal ganglia neurocircuitry in the pathology of psychiatric disorders. Psychiatry Clin. Neurosci..

[94] Sheffield J.M., Huang A.S., Rogers B.P., Giraldo-Chica M., Landman B.A., Blackford J.U., Heckers S., Woodward N.D. (2020). Thalamocortical Anatomical Connectivity in Schizophrenia and Psychotic Bipolar Disorder. Schizophr. Bull..

[95] Nortje G., Stein D.J., Radua J., Mataix-Cols D., Horn N. (2013). Systematic review and voxel-based meta-analysis of diffusion tensor imaging studies in bipolar disorder. J. Affect. Disord..

[96] Versace A., Almeida J.R., Hassel S., Walsh N.D., Novelli M., Klein C.R., Kupfer D.J., Phillips M.L. (2008). Elevated left and reduced right orbitomedial prefrontal fractional anisotropy in adults with bipolar disorder revealed by tract-based spatial statistics. Arch. Gen. Psychiatry.

[97] Ching C.R.K., Hibar D.P., Gurholt T.P., Nunes A., Thomopoulos S.I., Abé C., Agartz I., Brouwer R.M., Cannon D.M., de Zwarte S.M.C. (2022). What we learn about bipolar disorder from large-scale neuroimaging: Findings and future directions from the ENIGMA Bipolar Disorder Working Group. Hum. Brain Mapp..

[98] Magioncalda P., Martino M. (2022). A unified model of the pathophysiology of bipolar disorder. Mol. Psychiatry.

[99] Anticevic A., Savic A., Repovs G., Yang G., McKay D.R., Sprooten E., Knowles E.E., Krystal J.H., Pearlson G.D., Glahn D.C. (2015). Ventral anterior cingulate connectivity distinguished nonpsychotic bipolar illness from psychotic bipolar disorder and schizophrenia. Schizophr. Bull..

[100] Saccaro L.F., Delavari F., Van De Ville D., Piguet C. (2024). Hippocampal temporal dynamics and spatial heterogeneity unveil vulnerability markers in the offspring of bipolar patients. Bipolar Disord..

[101] Murphy F., Nasa A., Cullinane D., Raajakesary K., Gazzaz A., Sooknarine V., Haines M., Roman E., Kelly L., O’Neill A. (2022). Childhood Trauma, the HPA Axis and Psychiatric Illnesses: A Targeted Literature Synthesis. Front. Psychiatry.

[102] Ruocco A.C., Reilly J.L., Rubin L.H., Daros A.R., Gershon E.S., Tamminga C.A., Pearlson G.D., Hill S.K., Keshavan M.S., Gur R.C. (2014). Emotion recognition deficits in schizophrenia-spectrum disorders and psychotic bipolar disorder: Findings from the Bipolar-Schizophrenia Network on Intermediate Phenotypes (B-SNIP) study. Schizophr. Res..

[103] Winton-Brown T.T., Fusar-Poli P., Ungless M.A., Howes O.D. (2014). Dopaminergic basis of salience dysregulation in psychosis. Trends Neurosci..

[104] Karcher N.R., Rogers B.P., Woodward N.D. (2019). Functional Connectivity of the Striatum in Schizophrenia and Psychotic Bipolar Disorder. Biol. Psychiatry Cogn. Neurosci. Neuroimaging.

[105] Magioncalda P., Martino M., Conio B., Escelsior A., Piaggio N., Presta A., Marozzi V., Rocchi G., Anastasio L., Vassallo L. (2015). Functional connectivity and neuronal variability of resting state activity in bipolar disorder--reduction and decoupling in anterior cortical midline structures. Hum. Brain Mapp..

[106] Ongur D., Lundy M., Greenhouse I., Shinn A.K., Menon V., Cohen B.M., Renshaw P.F. (2010). Default mode network abnormalities in bipolar disorder and schizophrenia. Psychiatry Res..

[107] Modinos G., Renken R., Ormel J., Aleman A. (2011). Self-reflection and the psychosis-prone brain: An fMRI study. Neuropsychology.

[108] Liang Y., Jiang X., Zhu W., Shen Y., Xue F., Li Y., Chen Z. (2020). Disturbances of Dynamic Function in Patients With Bipolar Disorder I and Its Relationship With Executive-Function Deficit. Front. Psychiatry.

[109] Mullin B.C., Perlman S.B., Versace A., de Almeida J.R., Labarbara E.J., Klein C., Ladouceur C.D., Phillips M.L. (2012). An fMRI study of attentional control in the context of emotional distracters in euthymic adults with bipolar disorder. Psychiatry Res..

[110] van Neerven T., Bos D.J., van Haren N.E. (2021). Deficiencies in Theory of Mind in patients with schizophrenia, bipolar disorder, and major depressive disorder: A systematic review of secondary literature. Neurosci. Biobehav. Rev..

[111] Anticevic A., Yang G., Savic A., Murray J.D., Cole M.W., Repovs G., Pearlson G.D., Glahn D.C. (2014). Mediodorsal and visual thalamic connectivity differ in schizophrenia and bipolar disorder with and without psychosis history. Schizophr. Bull..

[112] Jauhar S., Nour M.M., Veronese M., Rogdaki M., Bonoldi I., Azis M., Turkheimer F., McGuire P., Young A.H., Howes O.D. (2017). A Test of the Transdiagnostic Dopamine Hypothesis of Psychosis Using Positron Emission Tomographic Imaging in Bipolar Affective Disorder and Schizophrenia. JAMA Psychiatry.

[113] Duggirala S.X., Schwartze M., Pinheiro A.P., Kotz S.A. (2019). Interaction of emotion and cognitive control along the psychosis continuum: A critical review. Int. J. Psychophysiol. Off. J. Int. Organ. Psychophysiol..

[114] Yeatman J.D., Dougherty R.F., Myall N.J., Wandell B.A., Feldman H.M. (2012). Tract profiles of white matter properties: Automating fiber-tract quantification. PLoS ONE.

[115] Colby J.B., Soderberg L., Lebel C., Dinov I.D., Thompson P.M., Sowell E.R. (2012). Along-tract statistics allow for enhanced tractography analysis. NeuroImage.

[116] Osanai Y., Shimizu T., Mori T., Yoshimura Y., Hatanaka N., Nambu A., Kimori Y., Koyama S., Kobayashi K., Ikenaka K. (2017). Rabies virus-mediated oligodendrocyte labeling reveals a single oligodendrocyte myelinates axons from distinct brain regions. Glia.

[117] Hafeman D.M., Chang K.D., Garrett A.S., Sanders E.M., Phillips M.L. (2012). Effects of medication on neuroimaging findings in bipolar disorder: An updated review. Bipolar Disord..

[118] Szeszko P.R., Robinson D.G., Ikuta T., Peters B.D., Gallego J.A., Kane J., Malhotra A.K. (2014). White matter changes associated with antipsychotic treatment in first-episode psychosis. Neuropsychopharmacol. Off. Publ. Am. Coll. Neuropsychopharmacol..

[119] Kanaan R., Barker G., Brammer M., Giampietro V., Shergill S., Woolley J., Picchioni M., Toulopoulou T., McGuire P. (2009). White matter microstructure in schizophrenia: Effects of disorder, duration and medication. Br. J. Psychiatry J. Ment. Sci..

[120] Kafantaris V., Spritzer L., Doshi V., Saito E., Szeszko P.R. (2017). Changes in white matter microstructure predict lithium response in adolescents with bipolar disorder. Bipolar Disord..

[121] Ambrosi E., Chiapponi C., Sani G., Manfredi G., Piras F., Caltagirone C., Spalletta G. (2015). White matter microstructural characteristics in Bipolar I and Bipolar II Disorder: A diffusion tensor imaging study. J. Affect. Disord..

[122] Emsell L., Leemans A., Langan C., Van Hecke W., Barker G.J., McCarthy P., Jeurissen B., Sijbers J., Sunaert S., Cannon D.M. (2013). Limbic and callosal white matter changes in euthymic bipolar I disorder: An advanced diffusion magnetic resonance imaging tractography study. Biol. Psychiatry.

