# Supplementary material for: Distinguished Frontal White Matter Abnormalities Between Psychotic and Nonpsychotic Bipolar Disorders in a Pilot Study
Source: Brain Sci. 2025 Jan 23;15(2):108. doi: 10.3390/brainsci15020108 (PMC11853555; doi:10.3390/brainsci15020108)
Supplement: Supplementary file 1 [file brainsci-15-00108-s001.zip › Fig S1_legend.pdf]

**Fig S1. Scatterplots of RD values in TBSS-ROI based on registration using DTI-TK.**

Scatterplots shows age-related RD values of PBD, NPBD, and HC.

Positive aging slope was significant only in NPBD (solid blue line $y = 0.006x + 0.212$).

PBD constantly exhibited more increased RD than NPBD and HC except one subject.

PBD: psychotic bipolar disorder (red triangle); NPBD: nonpsychotic bipolar disorder (blue circle); HC: healthy control (black square); RD: radial diffusivity
