# Supplementary material for: Distinguished Frontal White Matter Abnormalities Between Psychotic and Nonpsychotic Bipolar Disorders in a Pilot Study
Source: Brain Sci. 2025 Jan 23;15(2):108. doi: 10.3390/brainsci15020108 (PMC11853555; doi:10.3390/brainsci15020108)
Supplement: Supplementary file 1 [file brainsci-15-00108-s001.zip › TableS1_Group effects in TBSS analyses based on registration using DTI-TK.pdf]

Table S1. Group effects in the TBSS analyses based on registration using DTI-TK.

PBD compared to HC

Findings	WM tracts
Increased MD ($p_{tfce\text{-}FWE} < 0.010$*)	The bCC, the left gCC, ACR extending to the SFG-WM
Increased AD ($p_{tfce\text{-}FWE} < 0.009$*)	The left PLIC
Increased RD ($p_{tfce\text{-}FWE} < 0.013$*)	The gCC, the bCC, and the left ACR and SCR extending to the SFG-WM

No change in FA was found in PBD compared to HC.

No changes in any DTI measures were found in PBD compared with NPBD or in NPBD compared with HC.

Age and gender were included as covariate. *Corrected for multiple comparisons across voxels.

TBSS: tract-based spatial statistics; DTI-TK: Diffusion Tensor Imaging Tool Kit

ACR: anterior corona radiata; AD: axial diffusivity; bCC: body of the corpus callosum; FA: fractional anisotropy; gCC: genu of the corpus callosum;

MD: mean diffusivity; NPBD: nonpsychotic bipolar disorder; PBD: psychotic bipolar disorder; PLIC: posterior limb of the internal capsule;

$p_{tfce\text{-}FEW}$：corrected p value for multiple comparisons across voxels by family-wise error (FWE) corrections using threshold-free cluster enhancement (TFCE);

RD: radial diffusivity; SCR: superior corona radiata; SFG: superior frontal gyrus
