# Supplementary material for: Distinguished Frontal White Matter Abnormalities Between Psychotic and Nonpsychotic Bipolar Disorders in a Pilot Study
Source: Brain Sci. 2025 Jan 23;15(2):108. doi: 10.3390/brainsci15020108 (PMC11853555; doi:10.3390/brainsci15020108)
Supplement: Supplementary file 1 [file brainsci-15-00108-s001.zip › TableS2_Results of TBSS-ROI analyses based on registration using DTI-TK_.pdf]

**Table S2. Results of the TBSS-ROI analyses based on registration using DTI-TK.**

| DTI measure | PBD mean (SD) | NPBD mean (SD) | HC mean (SD) | 3 groups ANOVA (*F*) | 3 groups ANOVA (*p*) Effect size ($\eta^2$) | 3 groups group x age (*p*) Effect size ($\eta^2$) | 3groups group x gender (*p*) Effect size ($\eta^2$) | PBD vs NPBD (*p*) Effect size [95% CI] | PBD vs HC (*p*) Effect size [95% CI] | NPBD vs HC (*p*) Effect size [95% CI] |
|---|---|---|---|---|---|---|---|---|---|---|
| **FA** | 0.516 (0.030) | 0. 557 (0.050) | 0.555 (0.022) | 7.388 | 0.002* 0.280 | 0.001* 0.212 | 0.162 0.089 | 0.015* *d* = 1.520 [-0.083, -0.007] | 0.002* *d* = 1.607 [-0.080, -0.015] | 1.000 *d* = 0.087 [-0.034, 0.029] |
| **AD** | 1.401 (0.041) | 1. 379 (0.040) | 1.372 (0.035) | 1.533 | 0.231 0.085 | 0.079 0.136 | 0.419 0.049 | 0.795 *d* =0 .571 [-0.027, 0.071] | 0.269 *d* = 0.754 [-0.013, 0.071] | 1.000 *d* = 0.183 [-0.034, 0.048] |
| **RD** | 0.559 (0.036) | 0.504 (0.054) | 0.501 (0.026) | 12.439 | 0.000* 0.390 | 0.004* 0.172 | 0.045* 0.131 | 0.002* *d* = 1.877 [0.020, 0.102] | 0.000* *d* = 2.11 [0.033, 0.103] | 1.000 *d* = 0.235 [-0.026, 0.042] |

Age and gender were included as covariate.    *Bonferroni corrected.

TBSS: tract-based spatial statistics; DTI-TK: Diffusion Tensor Imaging Tool Kit; DTI: diffusion tensor imaging

AD: axial diffusivity; FA: fractional anisotropy; NPBD: nonpsychotic bipolar disorder; PBD: psychotic bipolar disorder; RD: radial diffusivity
