# Supplementary figures and images for: Distinguished Frontal White Matter Abnormalities Between Psychotic and Nonpsychotic Bipolar Disorders in a Pilot Study
Source: Brain Sci. 2025 Jan 23;15(2):108. doi: 10.3390/brainsci15020108 (PMC11853555; doi:10.3390/brainsci15020108)

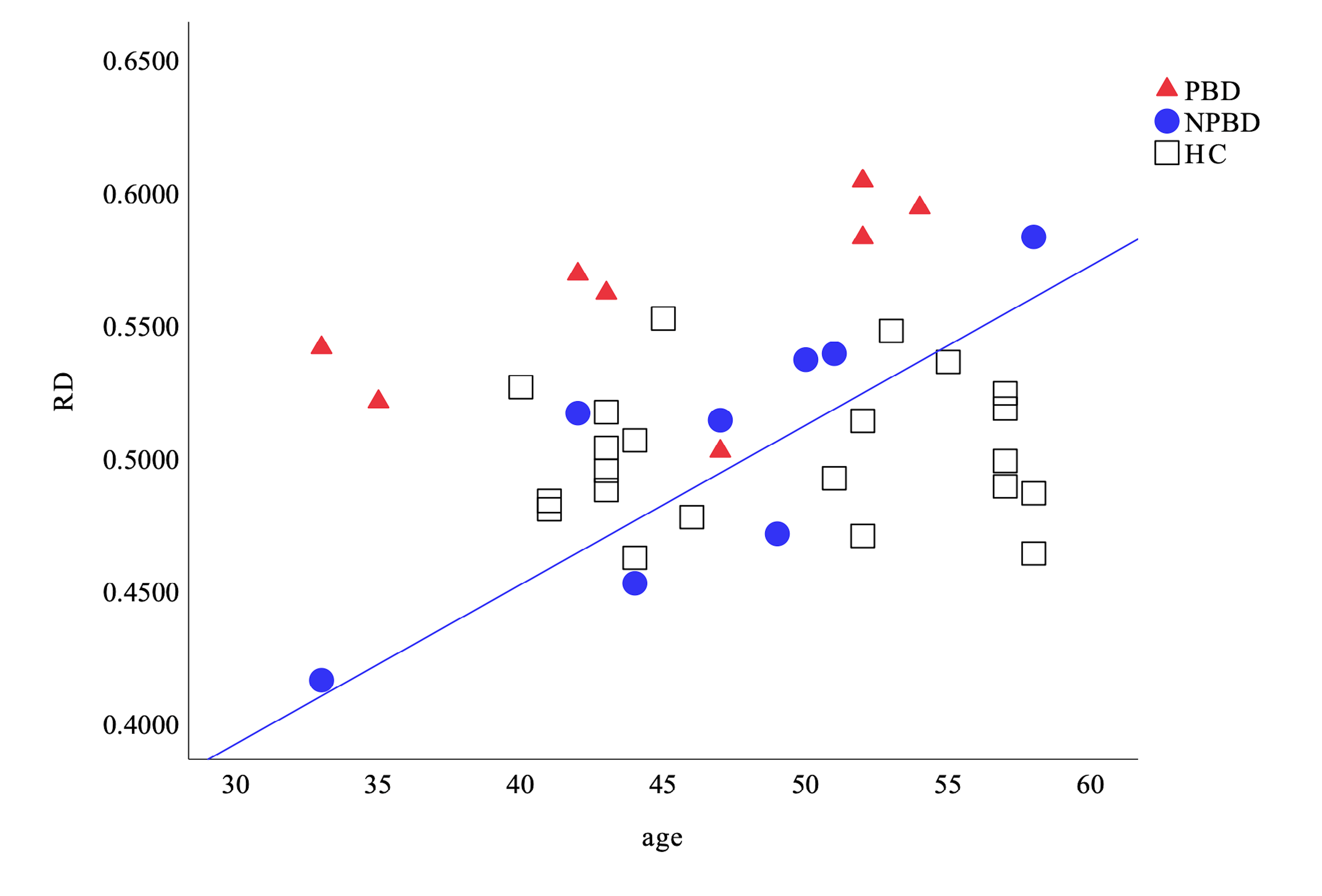

Supplement: Supplementary file 1 [file brainsci-15-00108-s001.zip › FigS1.png]
